# “Misuse” of RNA‐seq data in microbiome studies: A cautionary tale of poly(A)

**DOI:** 10.1002/mlf2.70012

**Published:** 2025-06-02

**Authors:** John Maringa Githaka

**Affiliations:** ^1^ Department of Biochemistry University of Alberta Edmonton Alberta Canada

The polyadenylation (poly(A)) in messenger RNA (mRNA) represents a well‐known and fundamental distinction between the host and the majority of microbiota. The increasing use of poly(A)‐enriched RNA sequencing (RNA‐seq) datasets to map out microbial abundances raises concerns that this approach may introduce bias, potentially skewing the results and leading to inaccurate conclusions. Here, I aim to raise awareness of this unintentional “misuse” of RNA‐seq data and present an analysis that demonstrates how such biases can arise. I also offer a simple framework to help researchers avoid these pitfalls, ensuring more accurate and reliable analyses of both host and microbiota RNA.

## PRELUDE: “INPUT DETERMINES OUTPUT”

In biological data acquisition, analysis, and interpretation, the adage “input determines output” exemplifies the need to ensure that the primary data are unbiased with respect to their intended use; otherwise, any subsequent bioinformatics analysis results will likely be flawed. The potential for flawed results is even higher in microbiome taxonomy research, where a single sample can contain from a few dozen to thousands of diverse microbiome species that need intricate delineation^1^. The quality of datasets used is essential for correctly identifying and classifying these species, while reliable abundance analysis is needed to understand their relative importance and interactions. Biased data in any of these areas can lead to incorrect interpretations of microbial roles, functions, and their impact on health, undermining the conclusions drawn from the research. Given the intricacy and variability of microbiomes, high‐quality data are essential for producing valid, reproducible, and meaningful results.

**Figure 1:**
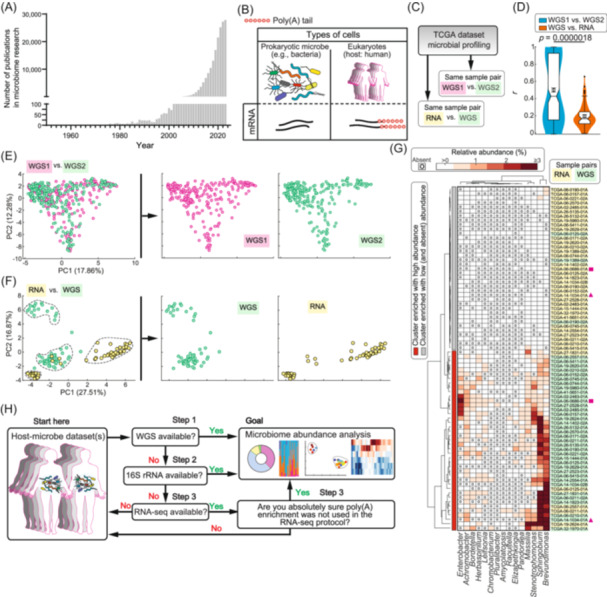
Microbiome studies, poly(A) tail differences, and the proposed framework for analysis. (A) Annual publications (from 1956 to 2023) associated with microbiome studies obtained by PubMed search (query “microbiome”). (B) Schematic of host and microbes, highlighting differences in poly(A) tail presence/absence. (C) Schematic for the two groups of sequencing pairs analyzed in microbial profiling of the TCGA dataset. Samples either sequenced twice by Whole genome sequencing (WGS) (i.e., WGS1 vs. WGS2) or RNA and WGS (i.e., RNA vs. WGS) were compared for microbial abundance concordance. Detailed custom codes to reproduce the subsequent analysis are deposited: https://github.com/maringa780/polyA. (D) Tukey boxplots of correlation coefficients of all the microbes per sample in each group of comparison. For each boxplot, constriction indicates the median, the notch indicates the 95% confidence interval, and box edges are 25th and 75th percentiles, while whiskers show extreme data points excluding “outliers” (black dots). Overlaid circle and error bars are the mean ± SEM. Background violin plot areas illustrate the kernel fitted probability density, that is, the proportion of the data at any point is represented by the width of the shaded area at that point. (E) Principal Component Analysis scatter plot of the TCGA genera abundances from Sepich‐Poore et al.'s work published in *Oncogene* (2024)^3^ using WGS1 vs. WGS2 samples (left), and scatter plots for each sequence type, shown to highlight individual sample distributions that might be masked when both groups are overlaid (right). Note that both WGS1 and WGS2 of the same samples show a very similar distribution pattern, indicative of high concordance in microbial abundances between them. (F) Similar plots to (E) for RNA vs. WGS samples. The platform type‐dependent clustering of the same samples (dotted boundaries) is indicative of high discordance between microbial abundances of WGS and RNA. (G) Unsupervised hierarchical clustering dendrograms and heatmap of microbial abundances from the top 15 discordant genera (listed in columns) between WGS and RNA (sample IDs are listed in rows) in the “TCGA‐GBM“ study. Euclidean distance metric and Ward's linkage algorithm were used to compute the dendrograms as detailed before^4^, ^5^. The clear enrichment of RNA and WGS samples in clusters corresponds with low and high microbial abundances, respectively. Sample pairs referenced in the main text are highlighted by ▲ and ■ symbols. (H) Proposed framework for microbiome abundance analysis on host–microbe datasets of interest.

The microbiome has captivated researchers for decades^2^. It encompasses a diverse community of microorganisms living in and on our bodies, as well as in our environment^2^. This intricate ecosystem is primarily dominated by bacteria, in close association with archaea, fungi, and viruses, and plays a profound role in human health, ecology, and beyond^2^. The recent explosion in microbiome research has witnessed an exponential increase in publications in this field (Figure 1A), as researchers capitalize on current and past omics datasets to map out novel host–microbe landscapes. For decades, researchers have relied on targeted marker gene sequencing methods, predominantly utilizing 16S ribosomal RNA (16S rRNA) gene sequencing to differentiate bacterial taxa^6^. This is achieved by sequencing the gene's species‐specific hypervariable regions, which are individually flanked by conserved sequences, providing good taxonomic resolution at the genus level^6^. The advent of next‐generation sequencing (NGS) ushered in the age of whole‐genome sequencing (WGS), given the technique's ability to sequence total DNA of a chosen sample at unprecedented speed. This opened up avenues for shotgun WGS metagenomics, which involves “untargeted (‘shotgun’)” WGS “of all (‘meta’) microbial genomes (‘genomics’)” in a sample, allowing species‐level resolution in analysis of the taxonomy, strain, and functional potential of microbial communities^6^.

As NGS revolutionized the field of genomics, RNA‐seq became one of its most impactful applications, especially in the field of cancer biology. The recent discovery of a potential cancer specific microbiome (intratumoral microbiota) has drawn attention to previous datasets of sequenced tumors^7^. While targeted marker gene sequencing and untargeted WGS metagenomics have dominated taxonomic studies^6^, some studies seeking to map out this intratumoral microbiota have inadvertently used classic RNA‐seq datasets, drawing potentially flawed conclusions. This is due to a well‐established fact that unlike most host mRNA, the predominant bacteria microbes lack the mRNA polyadenylation tail (poly(A) tail, Figure 1B), a fundamental difference that is primarily used in enriching host mRNA in most sequencing protocols (see Mofayezi et al.'s recent review on this fundamental difference^8^). To illustrate the consequences of this methodological oversight, I present a cautionary example showing how misinterpretation of RNA‐seq data can lead to a distorted view of the host–microbe landscape, deviating significantly from the ground truth.

## mRNA POLY(A) Tail Enrichment In RNA‐SEQ: MICROBIOME ACHILLES’ HEEL

The central dogma of molecular biology encapsulates the fundamental process by which genetic information flows within a biological system. This concept outlines the sequential transfer of information from DNA to RNA to protein, forming the basis of gene expression and the functioning of all living organisms. Central to this gene expression is the transcription of DNA into mRNA, which acts as the template that conveys genetic information to the ribosomes for protein synthesis. Sequencing of this mRNA in total cellular RNA is quite challenging, as mRNA account for just 1%–5% of the total RNA, with the majority of RNA molecules being rRNAs. To circumvent this challenge, two methods are commonly used to enrich for mRNA: rRNA depletion and poly(A) tail enrichment^9^. In eukaryotic cells, the poly(A) tail, which is a stretch of adenine nucleotides added to the 3′ end of mRNA transcripts, enhances mRNA's stability, aids in nuclear export, and promotes translation^8^. Most studies in eukaryotic cells have relied on the poly(A) tail enrichment approach to selectively isolate mRNA transcripts for RNA‐seq^9^. Unlike the eukaryotic cells, prokaryotic cells like bacteria, which constitute most of the microbiome community mRNA, lack the poly(A) tail modification. In fact, when present in these cells, the poly(A) tail serves as a tag for degradation^8^. Indeed, best practices for transcriptomic investigations into prokaryotic cells’ mRNA recommend RNA‐seq from the rRNA depletion method and strongly discourage use of the poly(A) tail enrichment approach^9^. A similar approach has been recommended for “dual RNA‐seq of pathogen and host” samples^10^.

Recent reports suggest that it is possible to perform genus‐ and species‐level taxonomic profiling of the microbiome in a host sample using RNA‐seq reads^11^, ^12^, ^13^, ^14^, ^15^, ^16^, ^17^, ^18^. This is predicated on the fact that not all RNA‐seq reads map to the host reference genome, leading to the logical conclusion that some may originate from the host's microbiome. Discoveries on microbiome communities beyond the classic “gut microbiome” have sparked a “gold rush” to map out the microbiome associated with different disease states. While most have focused exclusively on reads from WGS datasets, some have utilized RNA‐seq datasets without giving much consideration to the mRNA enrichment methods used. For instance, The Cancer Genome Atlas (TCGA) provides one of the largest resources for probing over 11,000 cases of human cancers, spanning 33 cancer types^19^. As noted by the TCGA consortium, mRNA sequencing of all tumors samples was performed using a poly(A) enrichment RNA preparation, and only a limited number of cases had total RNA sequenced following rRNA depletion (https://www.cancer.gov/ccg/research/genome-sequencing/tcga/using-tcga-data/types). For example, Poor et al.'s work published in *Nature* (2020) and *Oncogene* (2024)^3^, ^20^ relied on some poly(A)‐enriched TCGA RNA‐seq datasets to map out the human tumor‐specific microbiome, potentially drawing biased conclusions. Interestingly, while others have raised technical concerns (issues with batch effect correction and database contamination with host sequences) in the Poore et al.'s work published in *Nature* (2020)^20^, none of the criticisms highlighted the use of poly(A)‐enriched mRNA‐seq^21^, ^22^. Similarly, Shin et al.'s work on the microbial landscape in chronic obstructive pulmonary disease also reanalyzed the poly(A)‐enriched RNA‐seq dataset, having been motivated by “previous studies” that “have found evidence of infections caused by various viruses and bacteria”^23^. However, four out of the five cited studies behind this motivation used RNA‐seq to map out viral reads that are known to be polyadenylated, with the fifth citation specifically advocating for the rRNA depletion method for studying bacterial transcriptomes.

## PROOF OF CONCEPT: WHERE POTENTIAL MEETS REALITY

In response to previous critiques of their work, Sepich‐Poore et al.'s work published in *Oncogene* (2024)^3^ provided a robust analysis of the cancer microbiome abundances in TCGA datasets. To this end, I leverage a unique cohort of samples from this analysis that have been double‐sequenced using either WGS (“WGS1 vs. WGS2”) or RNA‐seq and WGS (“RNA vs. WGS”) (Figure 1C) and delve into the comparative accuracy of microbiome abundance profiles derived from these two platforms. Data preprocessing, quality control, the host depletion pipeline, and taxonomic classification are thoroughly detailed in Sepich‐Poore et al.'s work published in *Oncogene* (2024)^3^. Information on the matched samples used, as well as the code to reproduce the analysis and plots presented here, is available at https://github.com/maringa780/polyA. Indeed, the highest correlations per sample were observed in “WGS1 vs. WGS2” microbiome abundances, with about half of these samples having a higher correlation than the top correlation (excluding outliers) of “RNA vs. WGS” samples (Figure 1D, *p* value = 0.00000018). Next, Principal Component Analysis (PCA) was used to assess microbial abundances' similarity and dissimilarity in each comparison. Samples double‐sequenced with WGS1 and WGS2 showed a similar pattern on the PCA scatter plot (Figure 1E), indicative of high similarity in these microbial profiles. In contrast, samples double‐sequenced with RNA‐seq and WGS revealed distinct sequencing‐platform‐specific clustering (Figure 1F), highlighting high dissimilarity in the microbial abundances of these platforms on the same samples. To uncover some of the most significant discrepancies in the microbiome abundance estimates in “RNA vs. WGS,” I computed unsupervised hierarchical clustering (dendrogram and heatmap) of microbial abundances from the top 15 discordant genera (Figure 1G). Similar to the PCA scatter plot, samples largely segregated into platform‐dependent clusters. Specifically, 92.3% (36/39) of WGS samples were in the high abundance cluster, in contrast to just 12.8% (5/39) in RNA‐seq samples (Figure 1G), in line with an underestimation of these microbes in poly(A)‐enriched RNA‐seq‐based microbe profiling. For example, WGS indicated enrichment of the genus *Brevundimonas* and *Enterobacter* in samples “TCGA‐14‐1034‐01A” and “TCGA‐06‐0686‐01A,” respectively; yet, RNA‐seq indicated that they were absent in the same samples (samples highlighted with ▲ and ■ in Figure 1G). Importantly, the discrepancy between WGS and RNA‐seq in microbial abundance analysis (as shown in the heatmap in Figure 1G) aligns with the central argument of this article: poly(A)‐selected mRNA libraries fail to capture certain microbes present in the original samples. As a result, crucial insights into microbial functions and health impacts may be missed, skewing research findings, compromising future reproducibility and validation of the results, and distorting overall conclusions. Clearly, while whole‐microbiome RNA‐seq (metatranscriptomics) offers exciting possibilities for microbiome research, especially when seeking to explore microbial functions in response to host environments, it is crucial to acknowledge its limitations in accurately quantifying microbial abundance. This analysis reminds us that the promise of new technologies must be tempered with an understanding of their inherent biases, ensuring that conclusions drawn from microbiome research are both comprehensive and scientifically sound.

It is also worth noting that the presence of some microbial species in poly(A)‐enriched RNA‐seq datasets might arise from possible contamination in the process of RNA preparation and sequencing. For example, Robinson et al.'s work suggested the presence of TCGA sequencing center‐specific bacterial species^24^. In addition, despite most bacterial mRNA lacking poly(A) tails, some bacteria species’ specific mRNA persists in poly(A) selected libraries independent of contamination^24^. While this may inspire others to analyze and draw microbiome abundance conclusions from such datasets, the hypothesis and analysis here assert that those conclusions will reflect a biased perspective of the purported microbial landscape.

## PROPOSED FRAMEWORK: IN DATA WE TRUST, BUT VERIFY BEFORE YOU QUANTIFY

Datasets in scientific endeavors serve as a treasure map that needs careful analysis; otherwise, one might just end up digging in the wrong place. The case that I have made above seeks to increase scientists’ awareness of the potential misuse of mRNA datasets in microbiome studies when generated using poly(A) enrichment RNA preparations. As such, researchers interested in delineating host–microbe data in samples should adopt a framework that seeks to use the most appropriate dataset(s) for their analysis (Figure 1H). Specifically, WGS datasets should be the primary source of host–microbe analysis (Step 1, Figure 1H), given its overwhelming advantages over targeted marker gene sequencing methods^6^. In the absence of WGS datasets, targeted marker gene sequencing datasets, such as16S rRNA, should be considered next (Step 2, Figure 1H). If neither WGS nor marker gene datasets are available, taxonomic analysis of RNA‐seq datasets should only be performed if it can be confirmed that poly(A) enrichment was not used in the RNA preparation protocol (Step 3, Figure 1H). Furthermore, when two or more appropriate sequencing datasets (e.g., WGS, targeted marker gene sequencing, or non‐poly(A) enrichment datasets such as rRNA depletion) are available for the same samples (Steps 1–3, Figure 1H), the most correlated microbes across these methods can be identified, aiding in the identification of microbial species most likely involved in the disease. Finally, in cases where the metadata on RNA preparation are missing, one should contact the original authors of the dataset of interest for clarity. Importantly, for microbial communities’ analysis, the RNA preparation method used on the dataset should be clearly stated to avoid confusion and increase confidence in the authors’ conclusion.
